# Health, disability and quality of life among trans people in Sweden–a web-based survey

**DOI:** 10.1186/s12889-016-3560-5

**Published:** 2016-08-30

**Authors:** Galit Zeluf, Cecilia Dhejne, Carolina Orre, Louise Nilunger Mannheimer, Charlotte Deogan, Jonas Höijer, Anna Ekéus Thorson

**Affiliations:** 1 Department of Public Health Sciences, Karolinska Institutet, Stockholm, Sweden; 2 Centre for Psychiatry Research, Department of Clinical Neuroscience, Karolinska Institutet, Stockholm, Sweden; 3 Gender Team, Centre for Andrology and Sexual Medicine, Karolinska University Hospital, Stockholm, Sweden; 4 Department of Health and HIV-prevention, The Swedish Federation for LGBTQ Rights (RFSL), Stockholm, Sweden; 5 Department of Learning Information Management and Ethics, Karolinska Institutet, Stockholm, Sweden; 6 The Public Health Agency of Sweden, Stockholm, Sweden; 7 Unit of Biostatistics, Institute of Environmental Medicine, Karolinska Institutet, Stockholm, Sweden

**Keywords:** Trans, Transgender, Trans experience, Self-rated health, Self-reported disability, Quality of life, Sweden, Gender identity, Gender expression, Gender dysphoria, Gender nonbinary, Transvestite, Legal gender recognition

## Abstract

**Background:**

Swedish research concerning the general health of trans people is scarce. Despite the diversity of the group, most Swedish research has focused on gender dysphoric people seeking medical help for their gender incongruence, or on outcomes after medical gender-confirming interventions. This paper examines self-rated health, self-reported disability and quality of life among a diverse group of trans people including trans feminine, trans masculine, and gender nonbinary people (identifying with a gender in between male of female, or identify with neither of these genders) as well as people self-identifying as transvestites.

**Methods:**

Participants were self-selected anonymously to a web-based survey conducted in 2014. Univariable and multivariable regression analyses were performed. Three backward selection regression models were conducted in order to identify significant variables for the outcomes self-rated health, self-reported disability and quality of life.

**Results:**

Study participants included 796 individuals, between 15 and 94 years of age who live in Sweden. Respondents represented a heterogeneous group with regards to trans experience, with the majority being gender nonbinary (44 %), followed by trans masculine (24 %), trans feminine (19 %) and transvestites (14 %). A fifth of the respondents reported poor self-rated health, 53 % reported a disability and 44 % reported quality of life scores below the median cut-off value of 6 (out of 10). Nonbinary gender identity (adjusted Odds Ratio (aOR) = 2.19; 95 % CI: 1.24, 3.84), negative health care experiences (aOR = 1.92; 95 % CI: 1.26, 2.91) and not accessing legal gender recognition (aOR = 3.06; 95 % CI: 1.64, 5.72) were significant predictors for self-rated health. Being gender nonbinary (aOR = 2.18; 95 % CI: 1.35, 3.54) and history of negative health care experiences (aOR = 2.33; 95 % CI: 1.54, 3.52) were, in addition, associated with self-reported disability. Lastly, not accessing legal gender recognition (aOR = 0.32; 95 % CI: 0.17, 0.61) and history of negative health care experiences (aOR = 0.56; 95 % CI: 0.36, 0.88) were associated with lower quality of life.

**Conclusions:**

The results of this study demonstrate that the general health of trans respondents is related to vulnerabilities that are unique for trans people in addition to other well-known health determinants.

## Background

Trans people are a heterogeneous group of people whose gender identity and/or expression differ from the sex assigned to them at birth (gender incongruence) [1]. In this context, sex refers to the bodily characteristics often believed to determine if a body is masculine or feminine, while gender identity refers to the intrinsic feeling of a person of being male, female or an alternative gender [2]. Further, gender expression refers to external manifestation of gender [3]. Trans people may include crossdressers/transvestites and other gender non-conforming persons [4]. In the context of this study, people self-identifying as transvestites generally use clothes and other attributes to express their gender differently from the sex assigned to them at birth. Some trans people are gender dysphoric, a distress caused by incongruence between the gender identity and assigned sex at birth. They might need a change of legal gender and gender-confirming medical interventions such as hormone therapy, hair removal, vocal training and/or gender reassignment treatment that may consist of breast augmentation, breast removal and/or genital surgeries [5]. Many trans people, however, do not need any medical interventions at all [6, 7]. Transgender people may have a male or female gender identity (trans binary), but they could also have a nonbinary gender identity, identifying in between male or female, or as neither of these gender categories [8]. A detailed list of trans-related terms and definitions, based on the current discourse of trans communities of Sweden, is presented in Table 1.

**Table 1:** Definitions of trans-related terms

**Gender identity –** the gender one identifies with, such as man, woman or an alternative gender.
**Gender expression –** the way one expresses gender by use of certain clothes, hairstyles, accessories, make-up and other attributes.
**Sex –** bodily characteristics usually thought to determine if a body is masculine or feminine. A person’s sex is often assigned at birth according to the bodily features of the baby.
**Legal gender/Legal sex** – the gender stated in legal documents, such as birth certificate and passport. Legal gender can, in some countries, be changed. Most countries only have two legal genders; male and female.
**Cisgender –** when a person’s gender identity and expression is in line with their sex assigned at birth, non-trans person.
**Transgender/Trans –** when a person’s gender identity and/or expression does not align with their sex assigned at birth.
**Gender incongruence –** when a person’s gender identity and/or expression does not align with their sex assigned at birth.
**Gender dysphoria –** distress that gender incongruence might cause.
**Transsexual person–** when a person’s gender identity differ from the sex assigned at birth, usually having a male or female gender identity.
**Transsexual background/Formerly transsexual –** when a person used to be transsexual, but have undergone gender-confirming health care and does not identify as trans anymore.
**Trans feminine –** a person who was assigned male at birth, identifying or presenting as female or feminine.
**Trans masculine –** a person who was assigned female at birth, identifying or presenting as male or masculine.
**Gender nonbinary/Intergender/Gender queer –** a person, regardless of sex assigned at birth, identifying outside of the gender binary, not being male or female, perhaps being in between genders or a whole other gender.
**Transvestite/Crossdresser –** a person, who expresses their gender differently from their sex assigned at birth, as a way of expressing their identity.
**Transsexualism –** a medical diagnosis needed in Sweden to access gender-confirming health care. In many countries the diagnosis is called gender identity disorder or gender dysphoria.
**Legal gender recognition –** the process in which a person’s legal gender is changed to align with the person’s gender identity.
**Gender-confirming/Gender-affirming health care –** medical treatments aiming to alter the bodily characteristics in order to better align with the person’s gender identity and ease gender dysphoria. Examples of gender-confirming health care include hormone treatments, hair removal, vocal training and surgery.
**Sex confirmation surgery –** surgeries aiming to change the body in different ways to align better with the person’s gender identity. Examples may include breast augmentation, breast removal (mastectomy), creating new genitals, removing gonads, etc.

Trans people may face health challenges related to their trans identity and may also share common health concerns with cisgender people (non-trans people) [9]. Many health concerns are directly and indirectly related to exposure to prejudice, discrimination and violence [10]. Others are related to health care access. Trans people often report experiences of health care services being inadequate and insensitive to their needs [11]. Limited access to gender-confirming health care for those who need it can have serious health consequences [12–14]. Lastly, poor mental health and elevated suicide risk are often reported health concerns among trans people [5, 9, 14, 15].

Several researchers emphasize the importance of capturing the diversity of gender identities and expressions among trans people in research since that makes an important precondition for specific health needs [16, 17], health risks [17, 18] and health outcomes [4]. Yet, this diversity is rarely captured in research.

The health situation among trans people in Sweden is understudied. Previous Swedish research has mainly focused on gender dysphoric people seeking medical help for their gender incongruence [19], or on outcomes after medical gender-confirming interventions and change of legal gender [5, 20–26]. Johansson et al. found that the majority of transsexual persons after sex reassignment therapy reported improved global functioning [26] while in another long-term follow-up cohort study, transsexual persons after sex reassignment surgery had an increased mortality and an increased risk of suicide attempts compared to population controls [5]. Furthermore, the general health situation for the broader trans population in Sweden remains unknown. The aim of the present study is therefore to fill this knowledge gap by analysing self-rated health, self-reported disability and overall quality of life among a Swedish population self-identifying as trans.

## Methods

### Study setting

In Sweden, adults can change their legal gender to one of the two categories male or female, after undergoing an approximately two years long assessment period at a gender clinic. Since the law was updated in 2013, there are no longer requirements for having to undergo neither sterilization nor genital surgery in order to change legal gender. When this study was conducted, gender dysphoric people who did not fill the diagnostic criteria for transsexualism F64.0 (ICD-10) [27], identifying instead as gender nonbinary, could not access gender-confirming health care. All medical care, including genital surgery and facial feminization surgery is financed by the national health care system. Legally, some important developments have occurred in recent years including a fairly new Discrimination Act, protecting against discrimination in many sectors, including health care, on the ground of gender identity or expression [28].

### Data collection

Participants were self-selected anonymously to a web-based survey, carried out September - November 2014. Participants were recruited by advertisements and email invitations by Qruiser, Scandinavia’s largest online community for lesbian, gay, bisexual, trans and queer (LGBTQ) people. LGBTQ organisations promoted the study via different social media channels and personal invitations to email lists and Facebook groups. Advertisements were also made via Google AdWords and through flyers that were distributed at selected specialised gender clinics.

The survey included 106 questions covering eight main themes: background questions; health; wellbeing; gender-confirming treatment; living habits; experience of victimization and social contacts; sexual life and quality of life. The majority of questions were multiple-choice and some questions could be answered with multiple alternatives. The questionnaire was developed by a research team composed by researchers from Karolinska Institutet and from the Public Health Agency of Sweden as well as experts from the Swedish Federation for LGBTQ rights (RFSL) and the Swedish Youth Federation for LGBTQ rights (RFSL Ungdom). The survey was pre-tested by six trans-identified individuals and revised accordingly.

### Study population

Individuals aged 15 years and above, who live in Sweden, speak Swedish and who identified as being or having been trans were eligible to participate in the study. By the end of the data collection, 1205 individuals had participated. Of these, 77 respondents were excluded since they did not fill the eligibility criteria, an additional 329 respondents never submitted their responses after completing (some parts of) the survey and 3 respondents were identified as duplicates (after reviewing the data and using the duplicate function on Stata version 13.1 statistical software). Thus, 796 respondents remained for the main analyses.

### Dependent variables

*Self-rated health* was measured by the question: “How would you assess your general health?” The variable was categorised into good (“Very good” and “Good”), poor (“Poor” and “Very poor”) and fair self-rated health.

*Self-reported disability* was defined by the answers: (1) “Yes, to some extent” or, (2) “Yes, to a high extent” to the question “Do you have any physical or mental condition that impairs your work ability or hinders you in your everyday life?”

*Quality of life* was measured by the question: “How would you rate your present quality of life on a scale from 0 to 10?” For the regression analysis, the variable was dichotomized at the sample median (≥6).

### Independent variables

The variable *trans experience* was composed of two check-all-that-apply questions about gender identity and about the terms that best describe respondents trans experience. The variable was then coded into four categories:

**Trans feminine**: this category includes individuals who self-identifies as feminine or women regardless of their legal gender status. Individuals were coded into this category if they exclusively checked the “Woman” alternative, the “Woman” and the “Queer” alternatives or who specified female gender identity in the comment field on the question on gender identity and who checked the alternatives “Transgender”, “Transsexual”,”Formerly transsexual” or specified a feminine trans identity in the comment field. Since most research concerning trans people has focused on trans feminine individuals [17], we used trans feminine respondents as the reference group in the statistical analysis.

**Trans masculine**: this category includes individuals who self-identifies as masculine or men regardless of their legal gender status. Individuals were coded into this category if they exclusively checked the “Man” alternative, the “Man” and the “Queer” alternatives or who specified male gender identity in the comment field on the question on gender identity and who checked the alternatives “Transgender”, “Transsexual”,”Formerly transsexual” or specified a masculine trans identity in the comment field.

**Gender nonbinary**: this category includes individuals who did not self-identify with a man or woman category. Individuals were coded into this category if they did not fill the criteria to fit the other categories and if they chose both the alternatives “Woman” and “Man”, the alternative “Both man and woman/in between man and woman”, “Queer”, “None/neither man nor woman”, “Unsure” or by indicating a nonbinary gender identity in the comments field on the question on gender identity. In addition, individuals checked the alternative “Intergender”, the combination alternatives “Transgender” and “Intergender”, “Transsexual” and “Intergender”, “Formerly transsexual” and “Intergender”, or by indicating a nonbinary trans identity in the comment field.

**Transvestites**: This group includes individuals who exclusively checked the alternatives “Transvestite”, both the alternatives “Transgender” and “Transvestite” or by indicating being transvestite in the comments field and could check any of the alternatives on the gender identity question.

Respondents who submitted contradicting responses were carefully examined with regards to other items such as change of legal gender, in order to code them into a category of trans experience.

Sociodemographic information was collected including age, country of birth (Sweden or other), county, employment status, education and income. *Age* was collapsed into five categories representing different age groups, extracted from participants reported year of birth. *Counties* were dichotomised into small (<500,000 inhabitants) and large (>1,000,000 inhabitants) counties. *Income* was self-reported as net income per month in Swedish crown currency, here presented in Euro. An average monthly net income in Sweden for an employee within the public sector is approximately 2570 Euro. The variable *employment status* was gathered by the check-all-that-apply question “What is your main source of income?” Respondents who selected the alternatives “Work” or “Student loan” were categorised as ‘working/studying’. Respondents were categorised as ‘unemployed/long-term sick leave’ if they selected the alternatives “Unemployment benefits”, “Sickness benefits” or “Income support” and if they did not fit the ‘working/studying’ category. Respondents were categorised as ‘retired’ if they selected the alternative “Pension” and if they did not select any of the responses to fit the former categories. Lastly, respondents were categorised as ‘other’ if they select the response “Other” and did not fit any of the other categories. The variable *Education* was based on the question “What is your highest level of education?”. The variable was collapsed into three categories: (1) ‘No high school education’, based on the responses “Ground school” or “2-years high school education”; (2) ‘Upper-secondary education or some university’, based on the alternatives “3-4 years high school education” or “University education equivalent to or less than 2.5 years”; and (3) ‘University education ≥ 3 years’, based on the response “University education for 3 or more years”.

The variable *history of negative health care experiences* was dichotomised from multiple responses to the question: “Have you experienced any/some of the following events when you encountered the health care system? Different events may have occurred in various health care settings”. Respondents who checked the alternative “I have received good treatment and good help” were coded into not having a history of negative health care experiences (history of negative health care experiences = ‘no’). Respondents were coded into having a negative health care experience (history of negative health care experiences = ‘yes’) if they checked one or more of the nine alternatives describing negative experiences including “I felt badly treated because I am trans” or “I needed to educate my health care providers on my trans identity in order to get appropriate help”.

The variable *tobacco use ever* was extracted from two questions on cigarette smoking or on use of snuff, which is a common mean of tobacco consumption in Sweden: (1) “Do you or have you ever used to smoke cigarettes?”, and (2) “Do you or have you ever used to use snuff?” The response alternatives were: (a) “No, I never smoked” (or used snuff); (b) “Yes, but I quit”; or (c) “Yes”. Respondents who replied “Yes” or “Yes, but I quit” to any of the two questions were considered to ever use tobacco. *Risk consumption of alcohol* was measured by the question “How many drinks (examples provided) do you have on a typical day when you drink alcohol?” Respondents who checked that they drink more than 5 drinks were defined to have risk consumption of alcohol. In addition, information was collected regarding illicit drug use six months prior to the study.

Other independent variables that were included in the analyses are religiosity, change of legal gender and openness with trans identity. *Religiosity* was defined by the responses “Religion affects my everyday life very much” or “Religion affects my everyday life quite a lot” to the question “Religion affects our life differently. How is it for you?”. *Change of legal gender* was based on the question “Have you changed your legal gender?”. The response alternatives were: (1) “Yes”, (2) “No, and I do not need to”, (3) “No, but I would like to”, (4) “No, I cannot change my legal gender since my desired legal gender is not available in Sweden today” (desired gender that does not fit the male/female categories), and (5) “Do not want to answer”. *Openness with being trans* was defined by the question “To what extent are you open with the fact that you are trans?”

*Practical support* was assessed by the question “Can you get any help from someone if you have a practical problem or are ill? For example get advice, borrow things, get help with grocery shopping, repairs, etc.”. *Social support* was assessed by the question “Do you have someone you can share your innermost feelings with and confide in?”. The questionnaire (in Swedish) is available as an appendix to the published report of the material elsewhere [29].

### Data analysis

Stata version 13.1 statistical software was used for the analysis. Descriptive statistics (frequencies) were carried out to describe the characteristics of study participants. Univariable and multivariable analyses were performed for each respective health outcome (i.e. self-rated health, self-reported disability and quality of life) against all variables of interest. For the outcome self-rated health, ordinal logistic regression was used and for the outcomes quality of life and self-reported disability logistic regression was used. The measures of association are presented as crude and adjusted odds ratios, with 95 % confidence intervals. Three backward selection regression models were performed, one for each health outcome, in order to identify the significant variables for each respective outcome. Due to missing observations on multiple items the regression models for the outcome self-rated health include 669 observations while self-reported disability and quality of life include 667 observations respectively. A Wald’s test was used for variables with more than one category in univariable and multivariable analyses, in order to assess significance of the variable as a whole.

## Results

A total of 796 individuals did self-recruit and participate in the study. Respondents were mainly born in Sweden (92 %), represented all 21 counties across Sweden and ranged in age from 15 to 94 years (mean age 33.3). Respondents represented a heterogeneous group with regards to trans experience, with the majority being gender nonbinary (44 %), followed by trans masculine (24 %), trans feminine (19 %) and transvestites (14 %). Frequencies of respondents’ characteristics are presented in Table 2.

**Table 2:** Key characteristics of study respondents and other explanatory variables (*n* = 796)

Characteristic	*n* (%)
Trans experience	
Trans feminine	149 (19)
Trans masculine	187 (24)
Gender nonbinary	346 (44)
Transvestite	112 (14)
Assigned sex at birth	
Woman	388 (49)
Man	360 (45)
Age categories	
15–19	82 (10)
20–29	342 (43)
30–44	202 (25)
45–64	130 (16)
65–94	39 (5)
Country of birth	
Sweden	729 (92)
Other than Sweden	67 (8)
County	
Large county	504 (65)
Small county	269 (35)
Employment status	
Working/studying	560 (72)
Unemployed/long-term sick leave	156 (20)
Retired	34 (4)
Other	33 (4)
Education	
No high school education	150 (19)
Upper-secondary education, or some university	400 (50)
University education ≥ 3 years	230 (29)
Other	16 (2)
Monthly net income (€)	
0–1416	425 (54)
1417–2125	98 (12)
2126–3238	145 (18)
>3239	80 (10)
History of negative health care experiences	
No	231 (31)
Yes	511 (69)
Tobacco use ever	
No	364 (47)
Yes	418 (53)
Alcohol risk consumption	
No	630 (80)
Yes	153 (20)
Illicit drug use past six months	
No	696 (90)
Yes	65 (8)
Do not want to answer	12 (2)
Religiosity	
No	717 (90)
Yes	78 (10)
Change of legal gender	
No, and do not need to	157 (20)
Yes	114 (14)
No, but would like to	298 (38)
No, and cannot change legal gender because the desired gender is not available in Sweden today	207 (26)
Do not want to answer	15 (2)
Openness with trans identity	
Always open	140 (18)
Sometimes open	406 (52)
Rarely open	137 (18)
Never open	51 (7)
Trans identity shows	37 (5)
Practical support	
Always	320 (41)
Most of the time	341 (44)
Almost never	87 (11)
Never	28 (4)
Social support	
Yes	572 (74)
No	205 (26)

### Self-rated health

Half of the respondents (51 %) reported good health and about a fifth (18 %) reported poor health. Odds ratios of crude and adjusted associations between explanatory variables and self-rated health are presented in Table 3.

**Table 3:** Factors associated with poor self-rated health (*n* = 669)

Variable	Good health *n* (%)	Fair health *n* (%)	Poor health *n* (%)	Crude odds ratios (95 % CI)	Adjusted odds ratios (95 % CI)
Trans experience					
Trans feminine	75 (50)	44 (30)	30 (20)	1.00 (Reference)	
Trans masculine	103 (55)	59 (32)	25 (13)	0.76 (0.50–1.16)	0.99 (0.61–1.63)
Gender nonbinary	140 (40)	122 (35)	84 (24)	1.42 (0.99–2.05)	2.19** (1.24–3.84)
Transvestite	88 (79)	19 (17)	5 (4)	0.26 (0.15–0.44)	0.92 (0.37–2.26)
Age categories					
15–19	30 (37)	36 (44)	16 (19)	1.00 (Reference)	–
20–29	147 (43)	116 (34)	79 (23)	0.91 (0.58–1.40)	
30–44	109 (54)	57 (28)	36 (18)	0.60 (0.37–0.96)	
45–64	91 (70)	27 (21)	12 (9)	0.29 (0.17–0.50)	
65–94	28 (72)	8 (20)	3 (8)	0.26 (0.12–0.58)	
Country of birth					
Sweden	371 (51)	222 (30)	136 (19)	1.00 (Reference)	–
Other than Sweden	35 (52)	22 (33)	10 (15)	0.90 (0.56–1.44)	
County					
Large county	257 (51)	153 (30)	94 (19)	1.00 (Reference)	–
Small county	136 (50)	85 (32)	48 (18)	0.99 (0.75–1.32)	
Employment status					
Working/studying	325 (58)	162 (29)	73 (13)	1.00 (Reference)	
Unemployed/long-term sick leave	39 (25)	58 (37)	59 (38)	4.07 (2.89–5.72)	2.59*** (1.70–3.97)
Retired	24 (71)	7 (20)	3 (9)	0.58 (0.27–1.23)	1.16 (0.40–3.34)
Other	14 (42)	10 (30)	9 (27)	2.09 (1.07–4.06)	1.33 (0.61–2.88)
Education					
No high school education	65 (43)	52 (35)	33 (22)	1.22 (0.86–1.74)	–
Upper-secondary education, or some university	195 (49)	129 (32)	76 (19)	1.00 (Reference)	
University education ≥ 3 years	139 (60)	59 (26)	32 (14)	0.63 (0.46–0.87)	
Other	7 (44)	4 (25)	5 (61)	1.46 (0.56–3.82)	
Monthly net income (€)					
0–1416	153 (36)	161 (38)	111 (26)	2.71 (1.74–4.22)	1.80* (1.08–2.99)
1417–2125	61 (62)	22 (23)	15 (15)	1.00 (Reference)	
2126–3238	98 (68)	36 (25)	11 (7)	0.72 (0.42–1.22)	0.88 (0.48–1.63)
>3239	70 (88)	8 (10)	2 (2)	0.21 (0.10–0.47)	0.43 (0.17–1.07)
Do not want to answer	22 (50)	16 (36)	6 (14)	1.45 (0.73–2.89)	0.89 (0.36–2.16)
History of negative health care experiences					
No	166 (72)	45 (19)	20 (9)	1.00 (Reference)	
Yes	214 (42)	176 (34)	121 (24)	3.50 (2.51–4.86)	1.92** (1.26–2.91)
Tobacco use ever					
No	189 (52)	110 (30)	65 (18)	1.00 (Reference)	–
Yes	209 (50)	130 (31)	79 (19)	1.22 (0.91–1.62)	
Alcohol risk consumption					
No	329 (52)	188 (30)	113 (18)	1.00 (Reference)	–
Yes	71 (46)	53 (35)	29 (19)	1.20 (0.86–1.67)	
Illicit drug use past six months					
No	368 (53)	215 (31)	113 (16)	1.00 (Reference)	
Yes	20 (31)	23 (35)	22 (34)	2.56 (1.59–4.10)	2.29** (1.33–3.95)
Do not want to answer	5 (42)	1 (8)	6 (50)	3.03 (0.93–9.82)	6.27* (1.30–30.17)
Religiosity					
No	362 (51)	225 (31)	130 (18)	1.00 (Reference)	–
Yes	44 (56)	19 (24)	15 (19)	0.79 (0.36–1.72)	
Change of legal gender					
No, and do not need to	120 (76)	28 (18)	9 (6)	1.00 (Reference)	
Yes	67 (59)	31 (27)	16 (14)	2.32 (1.38–3.89)	2.25* (1.04–4.85)
No, but would like to	136 (46)	105 (35)	57 (19)	3.80 (2.48–5.81)	2.82** (1.48–5.37)
No, and cannot change legal gender because the desired gender is not available in Sweden today	73 (35)	73 (35)	61 (30)	6.17 (3.93–9.67)	3.06*** (1.64–5.72)
Do not want to answer	7 (47)	5 (33)	3 (20)	3.75 (1.35–10.41)	1.72 (0.52–5.60)
Openness with trans identity					
Always open	71 (51)	39 (28)	30 (22)	1.00 (Reference)	–
Sometimes open	200 (49)	133 (33)	73 (18)	0.97 (0.67–1.41)	
Rarely open	65 (47)	48 (35)	24 (18)	1.02 (0.65–1.59)	
Never open	37 (72)	8 (12)	6 (16)	0.38 (0.19–0.77)	
Trans identity shows	17 (46)	11 (30)	9 (24)	1.21 (0.61–2.40)	
Practical support					
Always	215 (67)	66 (21)	39 (12)	1.00 (Reference)	
Most of the time	140 (41)	128 (38)	73 (21)	2.70 (1.99–3.66)	2.40*** (1.68–3.43)
Almost never	25 (29)	38 (44)	34 (27)	4.13 (2.64–6.46)	2.57*** (1.47–4.48)
Never	14 (50)	6 (21)	8 (29)	2.43 (1.13–5.22)	4.74** (1.80–12.52)
Social support					
Yes	304 (53)	177 (31)	91 (16)	1.00 (Reference)	
No	92 (45)	62 (30)	51 (25)	1.49 (1.10–2.02)	1.64* (1.11–2.42)

* *p* < .05 ** *p* < .01 *** *p* < .001

Being gender nonbinary (adjusted Odds Ratio (aOR) = 2.19; 95 % CI: 1.24, 3.84) was significantly associated with poor self-rated health, compared to being trans feminine. History of negative health care experiences (aOR = 1.92; 95 % CI: 1.26, 2.91) was significant in predicting poor self-rated health. Having changed legal gender (aOR = 2.25; 95 % CI: 1.04, 4.85), wanting to change legal gender (aOR = 2.82; 95 % CI: 1.48, 5.37) and not being able to change legal gender because the desired gender it is not available in Sweden (aOR = 3.06; 95 % CI: 1.64, 5.72) were all significantly associated with poor self-rated health, compared to not needing to change legal gender. Lack of social support was significant in predicting poor self-rated health (aOR = 1.64; 95 % CI: 1.11, 2.42). In comparison to those always having practical support, those who mostly have (aOR = 2.40; 95 % CI: 1.68, 3.43), rarely have (aOR = 2.57; 95 % CI: 1.47, 4.48) or never have practical support (aOR = 4.74; 95 % CI: 1.80, 12.52) have reported significantly poorer self-rated health. Poor self-rated health was more common among those with lower income (aOR = 1.80; 95 % CI: 1.08, 2.99) and among those unemployed or on long-term sick leave (aOR = 2.59; 95 % CI: 1.70, 3.97), compared to those working or studying. Illicit drug use six months prior to the study was additionally associated with poor self-rated health (aOR = 2.29; 95 % CI: 1.33, 3.95).

### Self-reported disability

Approximately half of the respondents (53 %) reported having a disability due to a physical or mental condition that impairs their work ability or hinders their everyday lives to some extent. Odds ratios of crude and adjusted associations between explanatory variables and self-reported disability are presented in Table 4.

**Table 4:** Factors associated with self-reported disability (*n* = 667)

Variable	No disability	Disability	Crude odds ratios	Adjusted odds ratios
	*n* (%)	*n* (%)	(95 % CI)	(95 % CI)
Trans experience				
Trans feminine	74 (50)	75 (50)	1.00 (Reference)	
Trans masculine	93 (50)	93 (50)	0.98 (0.64–1.51)	0.97 (0.57–1.65)
Gender nonbinary	124 (36)	221 (64)	1.75 (1.19–2.59)	2.18*** (1.35–3.54)
Transvestite	79 (71)	33 (29)	0.41 (0.24–0.69)	0.85 (0.40–1.81)
Age categories				
15–19	33 (40)	49 (60)	1.00 (Reference)	–
20–29	135 (40)	205 (60)	1.02 (0.62–1.67)	
30–44	103 (51)	99 (49)	0.64 (0.38–1.08)	
45–64	79 (61)	51 (39)	0.43 (0.24–0.76)	
65–94	19 (49)	20 (51)	0.70 (0.32–1.52)	
Country of birth				
Sweden	338 (47)	389 (53)	1.00 (Reference)	–
Other than Sweden	32 (48)	35 (52)	0.95 (0.57–1.56)	
County				
Large county	234 (47)	268 (53)	1.00 (Reference)	–
Small county	124 (46)	145 (54)	1.02 (0.75–1.37)	
Employment status				
Working/studying	304 (54)	255 (46)	1.00 (Reference)	
Unemployed/long-term sick leave	29 (19)	126 (81)	5.17 (3.34–8.01)	3.51*** (2.05–6.00)
Retired	16 (47)	18 (53)	1.34 (0.67–2.68)	3.31** (1.33–8.24)
Other	16 (48)	17 (52)	1.26 (0.62–2.55)	0.73 (0.31–1.69)
Education				
No high school education	61 (41)	88 (59)	1.20 (0.82–1.77)	–
Upper-secondary education, or some university	182 (46)	217 (54)	1.00 (Reference)	
University education ≥ 3 years	122 (53)	108 (47)	0.74 (0.53–1.02)	
Other	5 (31)	11 (69)	1.84 (0.62–5. 40)	
Monthly net income (€)				
0–1416	136 (32)	288 (68)	2.11 (1.35–3.30)	1.55 (0.93–2.59)
1417–2125	49 (50)	49 (50)	1.00 (Reference)	
2126–3238	100 (69)	45 (31)	0.45 (0.26–0.76)	0.51* (0.28–0.94)
>3239	63 (79)	17 (21)	0.26 (0.13–0.52)	0.43* (0.19 –0.94)
Do not want to answer	21 (49)	22 (51)	1.04 (0.51–2.14)	1.15 (0.45–2.89)
History of negative health care experiences				
No	148 (64)	83 (36)	1.00 (Reference)	
Yes	190 (37)	319 (63)	2.99 (2.16–4.13)	2.33*** (1.54–3.52)
Tobacco use ever				
No	188 (52)	175 (48)	1.00 (Reference)	–
Yes	176 (42)	241 (58)	1.47 (1.10–1.95)	
Alcohol risk consumption				
No	294 (47)	335 (53)	1.00 (Reference)	–
Yes	71 (47)	81 (53)	1.00 (0.70–1.42)	
Illicit drug use past six months				
No	339 (49)	355 (51)	1.00 (Reference)	
Yes	14 (22)	51 (78)	3.47 (1.89–6.40)	3.29*** (1.61–6.72)
Do not want to answer	5 (42)	7 (58)	1.33 (0.42–4.25)	1.03 (0.24–4.29)
Religiosity				
No	34 (44)	43 (56)	1.00 (Reference)	–
Yes	336 (47)	380 (53)	1.21 (0.57–2.56)	
Change of legal gender				
No, and do not need to	99 (63)	58 (37)	1.00 (Reference)	–
Yes	55 (48)	59 (529	1.83 (1.12–2.98)	
No, but would like to	144 (49)	152 (51)	1.80 (1.21–2.67)	
No, and cannot change legal gender because the desired gender is not available in Sweden today	61 (29)	146 (71)	4.08 (2.62–6.34)	
Do not want to answer	8 (53)	7 (47)	1.49 (0.51–4.33)	
Openness with trans identity				
Always open	58 (42)	81 (58)	1.00 (Reference)	–
Sometimes open	182 (45)	224 (55)	0.88 (0.59–1.30)	
Rarely open	70 (51)	66 (49)	0.67 (0.41–1.08)	
Never open	32 (63)	19 (37)	0.42 (0.21–0.82)	
Trans identity shows	14 (38)	23 (62)	1.17 (0.55–2.47)	
Practical support				
Always	171 (53)	149 (47)	1.00 (Reference)	–
Most of the time	144 (42)	195 (58)	1.55 (1.14–2.11)	
Almost never	30 (34)	57 (66)	2.18 (1.33–3.57)	
Never	16 (57)	12 (43)	0.86 (0.39–1.87)	
Social support				
Yes	265 (46)	306 (54)	1.00 (Reference)	–
No	97 (48)	107 (52)	0.95 (0.69–1.31)	

* *p* < .05 ** *p* < .01 *** *p* < .001

Being gender nonbinary (aOR = 2.18; 95 % CI: 1.35, 3.54) was associated with self-reported disability, compared to being trans feminine. History of negative health care experiences (aOR = 2.33; 95 % CI: 1.54, 3.52) was a significant predictor of self-reported disability. Disability was more often reported by those who are unemployed or on long-term sick leave (aOR = 3.51; 95 % CI: 2.05, 6.00) and those who are retired (aOR = 3.31; 95 % CI: 1.33, 8.24), compared to those working or studying. Illicit drug use six months prior to the study was additionally associated with self-reported disability (aOR = 3.29; 95 % CI: 1.61, 6.72). Lastly, higher income was negatively associated with self-reported disability.

### Quality of life

Overall, 44 % of the respondents reported quality of life scores below the median cut-off value (=6) while the rest (56 %) reported quality of life scores above the median cut-off value. Odds ratios of crude and adjusted associations between explanatory variables and quality of life are presented in Table 5.

**Table 5:** Factors associated with lower quality of life (*n* = 667)

Variable	High quality of life	Low quality of life n (%)	Crude odds ratios	Adjusted odds ratios
	*n* (%)	*n* (%)	(95 % CI)	(95 % CI)
Trans experience				
Trans feminine	64 (43)	82 (57)	1.00 (Reference)	–
Trans masculine	84 (45)	102 (55)	0.94 (0.61–1.46)	
Gender nonbinary	161 (47)	179 (53)	0.86 (0.58–1.28)	
Transvestite	36 (35)	68 (65)	1.47 (0.87–2.47)	
Age categories				
15–19	55 (68)	26 (32)	1.00 (Reference)	
20–29	155 (46)	184 (54)	2.51 (1.50–4.19)	2.62** (1.37–4.98)
30–44	86 (43)	113 (57)	2.77 (1.61–4.79)	2.14* (1.07–4.30)
45–64	41 (33)	82 (67)	4.23 (2.32–7.69)	3.42** (1.50–7.78)
65–94	9 (25)	27 (75)	6.34 (2.61–15.40)	2.50 (0.39–15.98)
Country of birth				
Sweden	318 (45)	394 (55)	1.00 (Reference)	–
Other than Sweden	28 (42)	38 (58)	1.09 (0.65–1.82)	
County				
Large county	207 (42)	286 (58)	1.00 (Reference)	–
Small county	128 (49)	135 (51)	0.76 (0.56–1.03)	
Employment status				
Working/studying	214 (39)	335 (61)	1.00 (Reference)	
Unemployed/long-term sick leave	95 (62)	57 (38)	0.38 (0.26–0.55)	0.39*** (0.24–0.63)
Retired	8 (26)	23 (74)	1.83 (0.80–4.18)	1.32 (0.20–8.72)
Other	18 (55)	15 (45)	0.53 (0.26–1.07)	0.65 (0.26–1.61)
Education				
No high school education	77 (52)	70 (48)	0.81 (0.55–1.19)	–
Upper-secondary education, or some university	185 (47)	206 (53)	1.00 (Reference)	
University education ≥ 3 years	78 (35)	146 (65)	1.68 (1.19–2.35)	
Other	6 (37)	10 (63)	1.49 (0.53–4.19)	
Monthly net income (€)				
0–1416	230 (55)	190 (45)	0.48 (0.30–0.76)	–
1417–2125	36 (37)	61 (63)	1.00 (Reference)	
2126–3238	42 (31)	94 (69)	1.32 (0.76–2.28)	
>3239	16 (21)	61 (79)	2.25 (1.13–4.47)	
History of negative health care experiences				
No	63 (29)	156 (71)	1.00 (Reference)	
Yes	259 (51)	247 (49)	0.38 (0.27–0.54)	0.56* (0.36–0.88)
Tobacco use ever				
No	157 (43)	206 (57)	1.00 (Reference)	–
Yes	189 (46)	226 (54)	0.91 (0.68–1.21)	
Alcohol risk consumption				
No	270 (44)	344 (56)	1.00 (Reference)	–
Yes	68 (45)	84 (55)	0.96 (0.6–1.38)	
Illicit drug use past six months				
No	293 (42)	399 (58)	1.00 (Reference)	
Yes	44 (68)	21 (32)	0.35 (0.20–0.60)	0.41** (0.21–0.78)
Do not want to answer	6 (50)	6 (50)	0.73 (0.23–2.29)	1.26 (0.28–5.59)
Religiosity				
No	37 (50)	37 (50)	1.00 (Reference)	–
Yes	309 (44)	394 (56)	1.18 (0.55–2.52)	
Change of legal gender				
No, and do not need to	39 (26)	113 (74)	1.00 (Reference)	
Yes	24 (21)	89 (79)	1.27 (0.71–2.28)	1.62 (0.79–3.35)
No, but would like to	167 (57)	124 (43)	0.25 (0.16–0.39)	0.33*** (0.18–0.59)
No, and cannot change legal gender because the desired gender is not available in Sweden today	107 (53)	96 (47)	0.30 (0.19–0.48)	0.32*** (0.17–0.61)
Do not want to answer	8 (57)	6 (43)	0.25 (0.08–0.79)	0.61 (0.16–2.31)
Openness with trans identity				
Always open	49 (35)	90 (65)	1.00 (Reference)	–
Sometimes open	184 (45)	221 (55)	0.65 (0.43–0.97)	
Rarely open	69 (50)	68 (50)	0.53 (0.33–0.86)	
Never open	21 (41)	30 (59)	0.77 (0.40–1.50)	
Trans identity shows	21 (57)	16 (43)	0.41 (0.19–0.86)	
Practical support				
Always	86 (27)	233 (73)	1.00 (Reference)	
Most of the time	183 (54)	158 (46)	0.31 (0.22–0.44)	0.31*** (0.21–0.46)
Almost never	60 (69)	27 (31)	0.16 (0.09–0.27)	0.21*** (0.11–0.42)
Never	14 (52)	13 (48)	0.34 (0.15–0.75)	0.26* (0.09–0.74)
Social support				
Yes	224 (39)	346 (61)	1.00 (Reference)	
No	120 (59)	84 (41)	0.45 (0.32–0.62)	0.49** (0.31–0.77)

* *p* < .05 ** *p* < .01 *** *p* < .001

Wanting to change legal gender (aOR = 0.33; 95 % CI: 0.18, 0.59) and not being able to change legal gender because the desired gender it is not available in Sweden (aOR = 0.32; 95 % CI: 0.17, 0.61) were associated with lower quality of life, compared to not needing to change legal gender. History of negative health care experiences was a significant predictor of lower quality of life (aOR = 0.56; 95 % CI: 0.36, 0.88). Lack of social support was associated with lower quality of life (aOR = 0.49; 95 % CI: 0.31, 0.77). In comparison to those always having practical support, those who mostly have (aOR = 0.31; 95 % CI: 0.21, 0.46), rarely have (aOR = 0.21; 95 % CI: 0.11, 0.42) or never have (aOR = 0.26; 95 % CI: 0.09, 0.74) practical support have reported significantly lower quality of life. Being unemployed or on long-term sick leave (aOR = 0.39; 95 % CI: 0.24, 0.63) compared to working or studying and illicit drug use six months prior to the study (aOR = 0.41; 95 % CI: 0.21, 0.78) were also significant predictors of lower quality of life. Nevertheless, older age was associated with better quality of life.

Interestingly, the variable openness with trans identity was not found to be associated with self-rated health, self-reported disability or with quality of life.

## Discussion

This study fills an important gap in the literature about the wellbeing and general health of a diverse group of trans people, including gender nonbinary individuals, trans people not needing gender-confirming health care and transvestites. Our findings show that trans experience is an important determinant of self-rated health and self- reported disability among study participants. Moreover, our results demonstrate that lack of legal gender recognition and history of negative health care experiences due to trans-incompetence or transphobia in the health care system, are important predictors of worse self-rated health, increased self-reported disability and lower quality of life among study participants.

Our results support previous evidence that trans people constitute a vulnerable group that need to be a target for reducing health disparities. Nearly a fifth of the study respondents reported poor health, over half reported a disability and nearly half reported quality of life below the median cut-off value. To set the results of this study in context, among a sample of over 9000 Swedes, nearly two-thirds reported good health in comparison to half of the respondents in our study. In the same survey, approximately a quarter of the respondents reported having a chronic disease, long-lasting symptoms or disability while more than half of the trans people in our study reported having a disability [30].

This study succeeded in capturing the diversity of trans identities and trans experiences within the larger group of trans people. The categorisation of different trans experiences that was executed in this study revealed that health vulnerabilities vary with regards to the different trans identities and experiences. We found that a nonbinary gender identity was associated with poor self-rated health and self-reported disability. There is a gap in the literature concerning nonbinary trans people. However, similar findings are reported for people who define themselves as genderqueer. Budge and colleagues, for example, reported that genderqueer respondents have high clinical levels of depression and anxiety [31] and Harrison and colleagues found that genderqueer respondents report elevated levels of discrimination, compared to their trans counterparts [32]. Gender nonbinary individuals face additional challenges in a world that by default categorises people in a binary system of genders, for instance in legal documents, in the use of gendered pronouns and in physical environments such as public restrooms [31]. The minority stress among trans people has largely been explained by the added social stress of deviating from the gender norm - perhaps the strongest norm structure in our society [33]. Reasonably, the magnitude of this deviation and/or the magnitude of minority stress experienced among nonbinary may be even larger than among binary trans people, also affecting self-rated health and self-reported disability. Moreover, when this study was conducted, gender nonbinary individuals could not access gender-confirming health care in Sweden, which could also partially explain the increased prevalence of perceived ill health and disability in this group.

Interestingly, participants who identified as gender nonbinary composed the largest group among study respondents. ‘Genderqueer’ or ‘nonbinary’ gender identities outside of the binary of female and male identities are increasingly being recognized in legal, medical and psychological systems and diagnostic classifications in line with the emerging presence and advocacy of these groups of people. Population-based studies show a small percentage, but a sizable proportion in terms of raw numbers, of people who identify as gender nonbinary. This group remain marginalized and at risk of victimization and of minority stress as a result of discrimination [34]. With the increased recognition of this group, younger generations of trans people may have different experiences than former generations. In a qualitative study on sexual health among trans people in Sweden, a generational difference was observed; younger trans people described trans issues in terms of the right to health, the right to individual gender identity and expression and the right to have those rights respected [35]. The majority of the respondents in our study were young and could hence be more prone to express a nonbinary gender identity compared to participants in previous studies.

We found a positive association between worse quality of life and wanting to change legal gender as well as not being able to change legal gender because the desired gender is not available in Sweden. This finding suggests that quality of life among trans people is likely to improve with legal gender recognition.

Poor self-rated health was also associated with wanting to change legal gender and not being able to change legal gender because the desired gender is not available in Sweden. However, poor health was reported even among those who had changed their legal gender, although to a smaller extent. This finding points out that despite improved quality of life, trans people may still perceive their general health as poor even after a change of legal gender. Self-rated health has not yet been evaluated in this group, however this finding is in line with studies evaluating psychiatric health [36], and call for improved care of this population.

Legal gender recognition is, in the context of this study, a precondition for access to gender-confirming health care. Previous studies have found associations between access to gender-confirming health care and a variety of negative health outcomes including depression [12, 13], increased discrimination [37], suicide ideations and attempts [14] and lower quality of life [38]. Lack of legal gender recognition is nevertheless an indicator of binary gender categories being perceived insufficient as the only alternatives for legal gender, since gender nonbinary people in Sweden are unable to change legal gender to other genders than male or female.

Our results differ from other findings showing overall good health and quality of life among trans people. Weyers and colleagues, for example, found that transsexual women scored well on self-reported physical and mental health, compared to the general Dutch female population [39]. Similarly, Motmans and colleagues found that the quality of life of trans women was similar to that reported in the general Dutch female population [40]. These studies, however, exclusively included trans people who had undergone gender-confirming interventions or who were in the process of transitioning and hence did not include trans people who do not need medical interventions, could not access medical interventions, or who define themselves as transvestites. Kuhn and colleagues, in contrast, found that trans people, 15 years after sex reassignment surgery, reported significantly lower quality of life for the domains general health, role limitation, physical limitation and personal limitation compared to female controls [41].

A majority of the study respondents reported a history of negative health care experiences, including trans-incompetence among health care professionals and postponing seeking health care due to previous experiences of transphobia. Negative health care experiences were, in addition, found to be associated with poor self-rated health, self-reported disability and lower quality of life. Barriers in accessing health care among trans people is well documented in previous studies [17, 37, 42]. Cruz found that postponement in seeking health care among trans people is mainly caused due to experiences of discrimination [17].

While certain health determinants are unique for trans people others, including income and employment status, are common for cisgender and trans people alike [43–45]. However, even these common vulnerabilities might be elevated among trans people. Older age, which was associated with better quality of life in our study, could perhaps be a stronger predictor for trans people compared to the general population due to the increased vulnerability often reported among trans youth and young adults in particular with regards to exposure to victimization [46, 47].

Lack of practical and social support were found to be significant predictors of poor self-rated health and lower quality of life. Social support is an important resource and can have a stress-buffering effect [48] and was shown to be a significant factor for self-rated health in the general Swedish population [49]. Specifically for trans people, social support was previously found to be a significant predictor for maintaining a high quality of life [50]. While social support is beneficial in many ways it might also be more challenging for trans people to obtain. Previous studies report that trans people perceive less social support from family in comparison to their cisgender siblings [51] and perceive social or familial rejection to a high extent [52].

Not surprisingly, illicit drug use was found to be associated with poor self-rated health, self-reported disability and lower quality of life. Substance use is a well documented coping strategy among minority groups experiencing minority stress, a state of social stress experienced by stigmatized minority groups [33]. Different studies have shown elevated risks of substance use among trans people [53–55]. In comparison with these studies, the prevalence of illicit drug use among study respondents is low. However, a lack of comparable Swedish data is hindering us from making any conclusions on comparisons with the general population.

Lastly, openness with trans identity was not associated with any of the outcomes of this study. In contrast, Kosciw and colleagues found that openness with gender identity and sexual orientation were associated with increased victimization but also with higher self-esteem and decreased depression [56]. Cruz found that trans people who were open with their trans identity experienced more discrimination when seeking health care [17]. Thus, it seems that openness could have beneficial health outcomes or at times increase certain vulnerabilities that we could not detect in our findings.

### Strengths and limitations

Several limitations are important to consider in interpreting the findings of this study. While efforts were made to reach deep into different types of online networks of individuals identifying as trans, this is a convenience sample. Online surveys have the potential of eliminating several types of selection biases and have proven to be important in including hard to reach groups in health research [43]. Additionally, our online survey offered anonymity and privacy when answering, contributing to circumvent social desirability biasing the answers. There may be still bias related to the fact that the survey was only available in Swedish. People whose Swedish is not their native language were perhaps less likely to participate, which could partly explain the low participation rate of people born outside of Sweden. Since participants were mainly recruited through LGBTQ organisations, trans people who are not affiliated with such organisations might have had fewer chances to participate in the study. However, the survey was open to anyone accessing the website and study participants were encouraged to spread the link. In another effort to recruit participants via other channels, Google advertisements were used in addition to flyers distributed at selected gender clinics.

Another important limitation is the use of backward selection, which may have resulted in overestimated odds ratios and too narrow confidence intervals. There is little prior knowledge on the health outcomes among trans people that our study examined and backward selection is therefore an appropriate procedure. In order to minimize the risk of resulting with an overfitted complete model due to the large number of independent variables analysed, we chose to employ that method.

Our study also presents major strengths. The design and conduction of this study was carried out in close collaboration with actors from LGBTQ organisations, the Public Health Agency of Sweden and researchers within the academia. This collaboration ensures the quality and credibility of this study as well as the dissemination of the results to decision-makers and to organisations which reach trans communities with their work.

## Conclusions

The results of this study demonstrate that the general health of trans respondents is related to vulnerabilities that are unique for trans people and include nonbinary gender identity, history of negative health care experiences and not accessing legal gender recognition, in addition to well-known health determinants such as employment status, income, age and social support. These results suggest that strategies that eliminate discrimination against trans people and increase understanding of trans-related issues in health care context as well as increased access to legal gender recognition could improve the overall health and quality of life of trans people in Sweden.
