# Erratum to: Health, disability and quality of life among trans people in Sweden–a web-based survey

**DOI:** 10.1186/s12889-016-3735-0

**Published:** 2016-10-14

**Authors:** Galit Zeluf, Cecilia Dhejne, Carolina Orre, Louise Nilunger Mannheimer, Charlotte Deogan, Jonas Höijer, Anna Ekéus Thorson

**Affiliations:** 1 Department of Public Health Sciences, Karolinska Institutet, Stockholm, Sweden; 2 Centre for Psychiatry Research, Department of Clinical Neuroscience, Karolinska Institutet, Stockholm, Sweden; 3 Gender Team, Centre for Andrology and Sexual Medicine, Karolinska University Hospital, Stockholm, Sweden; 4 Department of Health and HIV-prevention, The Swedish Federation for LGBTQ Rights (RFSL), Stockholm, Sweden; 5 Department of Learning Information Management and Ethics, Karolinska Institutet, Stockholm, Sweden; 6 The Public Health Agency of Sweden, Stockholm, Sweden; 7 Unit of Biostatistics, Institute of Environmental Medicine, Karolinska Institutet, Stockholm, Sweden

## Erratum

In the publication of this article [1] it was brought to our attention that the last sentence on page 12 incorrectly states: “Legal gender recognition, in the context of this study, is a precondition for access to gender-confirming health care”. The correct sentence should read: “Gender-confirming health care, in the context of this study, is a precondition for access to legal gender recognition”.

This sentence has been updated in the original article.
